# An extensive (co-)expression analysis tool for the cytochrome P450 superfamily in *Arabidopsis thaliana*

**DOI:** 10.1186/1471-2229-8-47

**Published:** 2008-04-23

**Authors:** Jürgen Ehlting, Vincent Sauveplane, Alexandre Olry, Jean-François Ginglinger, Nicholas J Provart, Danièle Werck-Reichhart

**Affiliations:** 1 Institute of Plant Molecular Biology, Centre National de la Recherche Scientifique UPR 2357, Université Louis Pasteur, 28 rue Goethe, 67000 Strasbourg, France; 2 Department of Cell and Systems Biology, University of Toronto, 25 Willcocks Street, Toronto, ON M5S 3B2, Canada

## Abstract

**Background:**

Sequencing of the first plant genomes has revealed that cytochromes P450 have evolved to become the largest family of enzymes in secondary metabolism. The proportion of P450 enzymes with characterized biochemical function(s) is however very small. If P450 diversification mirrors evolution of chemical diversity, this points to an unexpectedly poor understanding of plant metabolism. We assumed that extensive analysis of gene expression might guide towards the function of P450 enzymes, and highlight overlooked aspects of plant metabolism.

**Results:**

We have created a comprehensive database, 'CYPedia', describing P450 gene expression in four data sets: organs and tissues, stress response, hormone response, and mutants of *Arabidopsis thaliana*, based on public Affymetrix ATH1 microarray expression data. P450 expression was then combined with the expression of 4,130 re-annotated genes, predicted to act in plant metabolism, for co-expression analyses. Based on the annotation of co-expressed genes from diverse pathway annotation databases, co-expressed pathways were identified. Predictions were validated for most P450s with known functions. As examples, co-expression results for P450s related to plastidial functions/photosynthesis, and to phenylpropanoid, triterpenoid and jasmonate metabolism are highlighted here.

**Conclusion:**

The large scale hypothesis generation tools presented here provide leads to new pathways, unexpected functions, and regulatory networks for many P450s in plant metabolism. These can now be exploited by the community to validate the proposed functions experimentally using reverse genetics, biochemistry, and metabolic profiling.

## Background

Cytochrome P450 monooxygenases, which catalyze substrate-, regio- and stereo-specific oxygenation steps in plant metabolism, have evolved to a huge superfamily of enzymes. Plant genome sequencing initiatives recently revealed 39 full-length P450 genes in *Chlamydomonas reinhartii*, 71 in the moss *Physcomitrella patens*, 246 in *Arabidopsis thaliana*, 356 in rice and 312 in *Populus trichocarpa *[1]. However, according to the most recent survey [2], only 41 of the 246 coding sequences in the *A. thaliana *genome have been associated with a specific biochemical function(s). The high complexity of the P450 superfamily as opposed to the relatively scarce information available on the functions of individual P450 enzymes was one of the surprises of the first sequenced plant genomes [3-5]. Assuming that P450 number and diversification in plants mirrors the evolution of chemical-, ecological- and bio-diversity, it points to an unexpectedly poor understanding of secondary metabolism, even in model plants. This led us to assume that an extensive analysis of P450 gene expression might actually be used to identify the metabolic networks, to highlight overlooked aspects of plant metabolism, and to reveal functions of "orphan" P450 enzymes.

An extensive and sustained annotation of the P450 genes in sequenced organisms, including plants, is being carried out and has been made publicly available on a University of Tennesse website maintained by David Nelson (Table 1). Annotation of *A. thaliana *P450 genes has also been curated and collated in other databases by different organizations (Table 1). They include comments on genomic, cDNA and protein sequences, genetic maps, phylogeny, function, available mutants and tissue-specific gene expression based on a boutique P450 gene microarray. On the other hand, information on the expression of individual P450 genes can be obtained from large scale digital gene expression databases. Also several large scale co-expression tools are available to compare the expression profile of a gene of interest with individual genes, or all genes available on the microarray [6-10] (Table 1).

**Table 1:** Internet resources referred to in this manuscript

Name used	Full name	Uniform resource locator (URL)
*P450 resources*		
Nelson	Cytochrome P450 homepage	
Schuler	Functional genomics of *Arabidopsis *P450s	
PlaCe	*Arabidopsis *cytochrome P450	
Krochko	P450s in plants	
*General gene information resources*		
TAIR	The *Arabidopsis *information resource	
MAtDB	MIPS *Arabidopsis thaliana *database	
TIGR	*Arabidopsis thaliana *genome project	
SIGnAL	T-DNA express: *Arabidopsis *gene mapping tool	
*Expression data resources*		
Genevestigator	*Arabidopsis thaliana *microarray database and analysis toolbox	
BAR	The bio-array resource for *Arabidopsis *functional genomics	
PRIMe	Platform for RIKEN metabolomics	
ATTED II	*Arabidopsis thaliana *trans-factor and cis-element prediction database	
*Pathway annotation resources*		
TAIR-GO	Gene Ontology annotations at TAIR	
AraCyc	AraCyc pathways at TAIR	
KEGG	KEGG orthology (KO) – *Arabidopsis thaliana*	
FunCat	MIPS functional catalogue	
AcyLipid	The *Arabidopsis *lipid gene database	
BioPathAt	Biochemical pathway knowledge database	

Such resources have been used as a starting point to create the comprehensive database, 'CYPedia' (see Availability and requirements section for URL), which combines large scale P450 (co-)expression data with functional annotation. In a first step, Affymetrix ATH1 microarray data were extracted from publicly available experiments to generate comprehensive gene expression matrices for all P450s. In a second step, correlation of the expression of each P450 gene with the expression of 4,130 selected and carefully re-annotated genes representative of plant metabolism was examined. Such a comparative analysis reveals highly complex and divergent expression patterns for the majority of P450s, and provides novel clues on P450 functions, related pathways, and corresponding regulatory networks. This paper describes the construction of the database, its content, and provides some examples of general and more specific information, which can be extracted from it.

## Results and Discussion

### P450 gene family information and expression data

A total of 271 P450s from *A. thaliana *are listed in the PlaCe Arabidopsis P450 database [11]. Using the corresponding locus identifiers (Atxgxxxxx) 227 genes were found to be represented on the Affymetrix ATH1 microarray represented by 216 probe sets (see Methods for details). A list of all P450 genes, the associated AGI loci, and the probe sets used can be found in Additional File 1 and at the 'CYPedia' homepage. A description of their biochemical function is also given (if known) and links to relevant publications as well as to information in external databases, such as 'MAtDB, 'TAIR', or 'SIGnAL' (Table 1). We retrieved normalized gene expression data for the selected probe sets from the 'Genevestigator Digital Northern' tool [10] covering more than 1,800 microarrays. Upon background correction, the mean intensity ratios of replicates from each experiment was placed in one of the following four categories: i) organ and tissue samples from wild type plants (compared to background levels), ii) stress treatment of wild type plants (compared to untreated control), iii) hormone, nutrient (deprivation), and other treatments (compared to control), and iv) mutant plants (compared to wild type samples).

### Organ and tissue-specific expression

**Figure 1:**
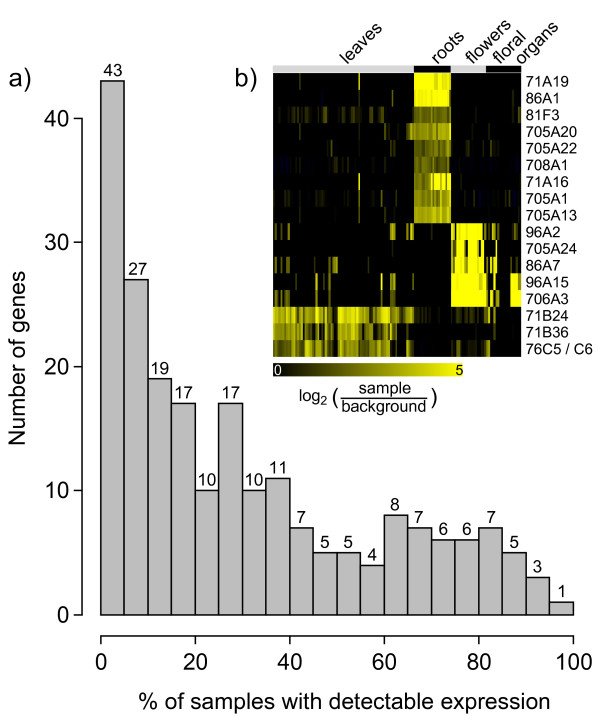
**Expression in the organ and tissue dataset**. Microarray data were retrieved from the Genevestigator database. Background was defined for each probe set as the mean intensity of all samples the probe set was called 'absent' (not significantly higher (p < 0.06) than the signal observed with the corresponding mismatch probe set). a) Histogram describing the frequency distribution of P450 genes expressed in the organ and tissue data set. Given in each bin is the number of probe sets representing P450 genes expressed more than twofold above background in 0% to 5%, 5% to 10%, etc., up to 95% to 100% of the 277 organ and tissue hybridization experiments. The number of genes in each bin is given on top of each bin. b) Genes that are expressed in more than 80% of root, whole flower, or leaf samples (>twofold above background), but not in more than 20% of all other samples (from a total of 277 samples) were selected. Shown are expression data of these genes in leaf, root, and flower samples as indicated on top. Expression intensities are compared to background (defined as the mean intensity of all samples called 'absent'

Across the organ and tissue data set, only seven P450 genes (represented by six probe sets) are not expressed more than twofold above background in any sample. An additional 6 genes (represented by 5 probe sets) are expressed in only one sample, and two genes in only two samples (Additional File 1). These may thus be considered as not detectably expressed in the organ sample set. This group includes all putative pseudogenes represented on the Affymetrix array. Conversely, 93 probe sets do show expression in more than two experiments, but in less than 20% of the 277 organ and tissue samples (Additional File 1; corresponding to the first four bins in Figure 1a), indicating highly specialized expression for 43% of the P450 genes represented on the array. Groups of flower, root, or leaf specific P450s are apparent. For example, 56 probe sets exhibit expression (twofold above background) in more than 80% of all root samples (23 experiments); of these, nine are expressed in less than 20% of other samples (Figure 1b). Using the same definition, we also identified five flower specific and four leaf specific P450s. These represent the most specifically expressed genes (Figure 1b). On the other hand, only 16 probe sets indicate expression in more than 80% of the tissue and organ samples covered (Additional File 1), and the corresponding 18 P450 genes may thus be considered constitutively expressed or house-keeping genes (last four bins in Figure 1a). The complete P450 organ and tissue expression matrix can be found at the 'CYPedia' web page following the link 'view matrices'.

**Figure 2:**
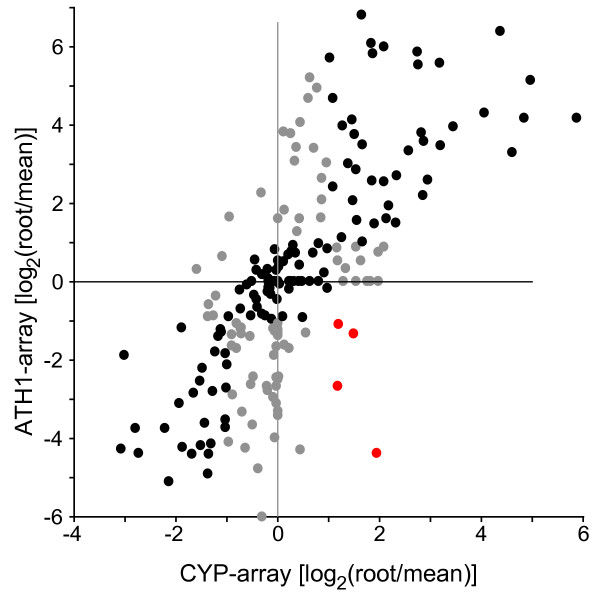
**Comparison of expression data between platforms**. P450 expression data generated using a spotted microarray covering gene specific PCR products (CYP-array) were retrieved from the 'Functional Genomics of Arabidopsis P450s' web page (Table 1). In this analysis, signal intensities in roots from 1 week old seedlings were generated by comparison to a 'universal RNA' sample [2]. Not detectable intensities were artificially set to a ratio of 0.05 compared to the 'universal control' and after log_2_-transformation expression data were mean centered across the experiments. Expression data from published Affymetrix ATH1 array hybridizations were processed as described in Methods. The mean intensities from 17 experiments derived from young roots were selected. To generate a control similar to the 'universal RNA', mean intensities from 69 experiments covering similar samples were calculated and log_2 _ratios were generated. Shown is a 2 × 2 plot comparing the mean centered expression ratios [log_2_(sample/mean)] from both platforms using data for all P450 genes represented on both array types. Data points following the same trend are shown in black, points which are more than twofold different from the average expression in one platform, but less than twofold different in the other are shown in gray. Red dots indicate genes with opposing expression using the two platforms

We compared expression of the highly specific genes with expression data generated using a dedicated P450 array generated by spotting gene specific PCR products [2]. Most organ specific genes identified here also show a predominant or exclusive expression in the respective organs using the boutique array (not shown). Also on a larger scale, the expression profile observed with the ATH1 array is in good agreement with results from the boutique array (Figure 2). We selected samples similar to those used on the boutique array from the Affymetrix organ data set and generated mean centered expression ratios from roots compared to the average expression in all organs analyzed. The majority of P450s follow the same trend in both array platforms with R^2^-values for a linear regression of 0.508 (Figure 2). Another group is ambiguous, as its expression is different from the average (more than twofold) using one platform, while the other suggests close to average expression. Only for four genes opposing results were obtained in the comparison of the two platforms. Although correlations were less pronounced in the other organ comparisons (data not shown), they also suggest a good agreement between the different methods, in particular given the large difference in the biological material used. The present analysis, however, benefits from a much larger set of experiments.

### Stress response

**Figure 3:**
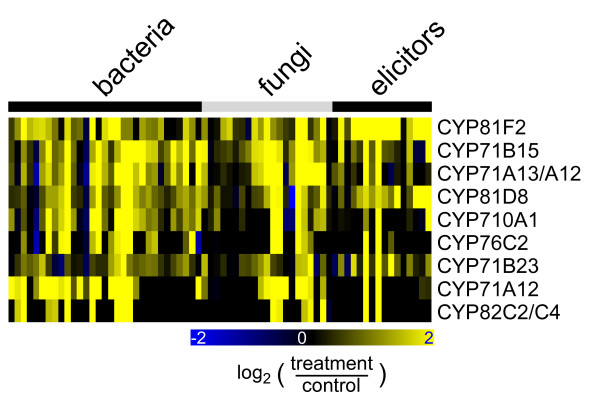
**Pathogen induced expression of selected P450s**. Microarray expression data were retrieved from the 'Genevestigator' database and processed as described in Methods. Selected genes that are up-regulated (>twofold) in more than 30% of at least one treatment group as indicated on top are shown. The complete set of genes fulfilling this criterion is shown in Additional File 2. Background corrected expression intensities were compared to untreated control experiments and log_2_-ratios were used for visualization. The resulting heatmap is color coded as indicated. Details on the individual samples can be found in Additional File 2.

A large group of P450s is responsive to one or several stresses across the 239 stress treatment experiments. More genes are up-regulated than down-regulated. While 38 probe sets show induction in more than 20 experiments, only two genes are repressed in more than 20 treatments. The complete stress response matrix of all P450s can be found at the 'CYPedia' web page following the link 'view matrices'. To highlight stress induction of P450s, we selected 49 probe sets representing 53 P450s showing more than twofold up-regulation in at least 30% of the experiments, within at least one of the treatment groups (Additional File 2). A group of nine probe sets representing eleven P450s stands out as being strongly induced by bacterial and fungal pathogens (Figure 3). These genes are induced rapidly in incompatible interactions between *A. thaliana *and *Pseudomonas syringae*, while induction in compatible interactions is comparatively slower as it has been observed for many defense related genes [12,13]. They are also induced by elicitors and by some abiotic stresses including oxidative, osmotic, and UV stress (Figure 3, Additional File 2). Among these genes, *CYP71B15 *has been well characterized as being pathogen-responsive and has been shown to encode an enzyme involved in the last step of camalexin biosynthesis, the major *A. thaliana *phytoalexin [14,15]. More recently, CYP71A13, was shown to catalyze an earlier step in camalexin formation [16]. Also previously characterized as differentially regulated in compatible and incompatible interactions and senescence is *CYP76C2 *[17], although in this case the protein function was not elucidated. Conversely, *CYP710A1 *had not been implicated in defense response, but was shown to be involved in stigmasterol biosynthesis [18]. So far, no function or involvement in defense has been described for the remaining genes in this group.

Another distinct cluster is defined by a group of 13 P450s (starting with *CYP74A *in Additional File 2). These genes are not (or weakly) responsive to pathogens, but are induced by several abiotic stresses, in particular by wounding, oxidative stresses (such as treatment with paraquat, ozone or H_2_O_2_), genotoxic stress (imposed by bleomycin), and by osmotic and salt stress (treatment with mannitol and NaCl, respectively). Within this group are the well characterized allene oxide synthase (AOS, CYP74A) and the hydroperoxyde lyase (HPL, CYP74B2) [19]. Both enzymes are involved in the oxylipin pathway leading to the biosynthesis of jasmonate and other oxygenated lipid derivatives involved in stress signaling. Also in this group is *CYP86A2*, which encodes an enzyme that ω-hydroxylates fatty acids and is involved in cuticle oxylipin metabolism [20,21].

### Hormone response

**Figure 4:**
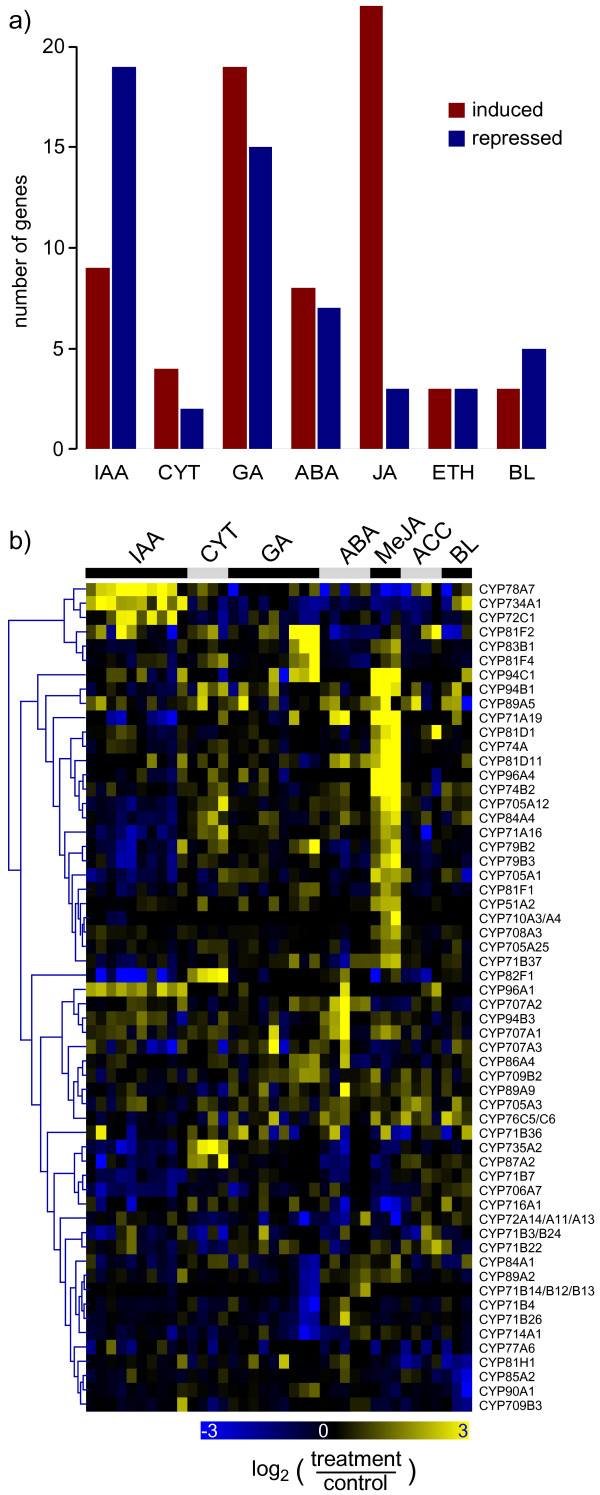
**Hormone responsive expression**. Microarray expression data were retrieved from the 'Genevestigator' database and processed as described in Material and Methods. Background corrected expression intensities were compared to untreated control experiments and log_2_-ratios were used. Genes that are up- or down-regulated (>twofold) in more than 30% of each treatment group as indicated were selected. a) Number of P450s which are responsive to each treatment. b) Hierarchical cluster analysis with complete linkage. The resulting heatmap is color coded as indicated.

Many P450s appear induced by treatment with methyl jasmonate (MeJ) (Figure 4). While 22 P450s are induced in more than 30% of all MeJ treatment experiments, only three are repressed (Figure 4a). Among the former are again *CYP74A *and *CYP74B2*, involved in the metabolism of fatty acid hydroperoxides [19], which are well known to be induced by jasmonate, but also a large number of additional P450s (Figure 4b). Not all these are expected to be involved in oxylipin metabolism, but the group may include genes involved in other pathways regulated by jasmonate. This holds true for CYP79B3, which converts tryptophan to the corresponding oxime, thus leading to the biosynthesis of indole glucosinolates, to camalexin, and to auxin [22-24]. It is interesting to note that *CYP79B3 *is repressed upon indole acetic acid (IAA) treatments. Other obvious groups comprise P450s that are strongly induced by IAA treatment (top of Figure 4b), or repressed by gibberellic acid (GA) in seeds (lower part of Figure 4b, starting with *CYP84A1*). In general, an extensive crosstalk between different hormone responses is apparent: eleven P450s are responsive to more than one hormone (> twofold) in at least three treatment experiments per hormone group. Antagonistic transcriptional responses of individual P450s are apparent between IAA and GA, MeJ and IAA, and cytokinin and IAA (Figure 4b). Strikingly, most of the hormone responsive P450s, when their functions are characterized, are themselves involved in hormone biosynthesis or catabolism: e.g. CYP734A1 (BAS1) and CYP72C1 (SOB7) are both involved in brassinosteroids catabolism [25,26], CYP735A2 is catalyzing *trans*-zeatin formation [27], and CYP79B2 is involved in IAA biosynthesis [24,28]. Other hormone-responsive P450s with so far uncharacterized functions may thus also participate in hormone metabolic networks.

### Mutant wild type comparisons

The mapping of P450 expression in mutants most often highlights very specific responses in isolated mutants or mutant groups. In a few cases only, subsets of ten or more genes are co-regulated in response to one or several mutations. Such coordinate responses provide leads to metabolic pathways as shown below. The most striking feature revealed by this data set is a very strong positive correlation of the activation of the set of P450 genes involved in stress response with the activation of the LEAFY gene [29]. The complete P450 mutant response matrix can be found at the 'CYPedia' web page following the link 'view matrices'.

In summary, expression matrices identify groups of genes with specific functions during plant development or roles in plant defense, and signaling networks. These may guide further investigation into the function of individual members of this large gene family, including fine expression analyses, description of mutant phenotypes and tissue-targeted metabolic profiling. Obvious hormonal networking and cross-talk may help to identify other enzymes involved in hormonal homeostasis and to highlight new and so far overlooked signaling pathways.

### Co-expression analysis

P450s catalyze slow and irreversible steps in all branches of the plant secondary metabolism. The underlying hypothesis of the CYPedia approach assumes that genes acting in the same biochemical pathway are co-expressed. When their function is known, P450s are usually co-regulated with other enzymes in the same branch-pathway [6,30]. Assuming that this may hold true also for yet uncharacterized P450s, we performed a comprehensive co-expression analysis comparing the expression of each P450 with that of 4,130 selected genes involved in *A. thaliana *metabolism. These were retrieved from diverse databases including 'KEGG', 'AraCyc, 'AcylLipid', BioPathAt', and selected publications devoted to the annotation of secondary metabolic pathways (Litpath) [30-35]. A list of all pathways and the associated genes can be found from the 'CYPedia' page following the link 'browse pathways'. For these genes, we then added annotations derived from the 'Functional Catalogue' at 'MatDB' [36] and manually curated 'GeneOntology' terms from 'TAIR' [37], as well as gene descriptions from 'TAIR' (Table 1). Based on a manual assessment of the combined annotations and literature reviews, each gene was given an annotation score reflecting the accuracy of the annotation (see Methods for details).

The annotation information of each gene was combined with expression data as described above for the P450 genes. Using the four expression vectors for each P450 as bait we calculated Pearson correlation coefficients (r-value) with each of the 4,130 selected genes for a total of 3.78 × 10^6 ^calculations on a Beowulf computer cluster. For each P450, similarly expressed genes (r > 0.5) were kept. Based on the number and annotation score of co-expressed genes, co-expressed pathways were identified for each P450 and expression dataset. The lists of co-expressed pathways can be found from the 'CYPedia' home page following the 'pathway maps' link for each P450. From there, links can be found to the individual heatmaps depicting the expression profile and detailed information of all co-expressed genes in each of the four data sets.

### Validation of pathway prediction: the phenylpropanoid metabolism as an example

In most cases, predicted functions based on top scoring co-expressed pathways agree well with the actual function of characterized P450s (Additional File 3). For 27 out of 43 P450s with known functions the correct pathway was predicted using this approach (63% success rate). For an additional four P450s, no co-expressed pathways were identified. This was in most cases because the gene was not expressed to detectable levels in any experiment. Of the eleven P450s for which a wrong pathway was predicted based on co-expression analysis, three had the correct pathway present within the ten highest scoring pathways. This leaves eight genes for which no correct pathway was identified (19% false identification rate). Most of those are involved in hormone metabolism.

Among the correctly predicted P450s are all three hydroxylases involved in lignin part of the phenylpropanoid pathway [38]. For example, when using *CYP73A5 *encoding cinnamate 4-hydroxylase (C4H) as bait, both in the organ and stress data sets all other genes characterized to act in the general phenylpropanoid pathway were retrieved with r-values higher than 0.5 (Additional File 4). Correlations were less pronounced in the remaining two datasets, but the annotated pathways 'Phenylpropanoid Metabolism' (BioPath) and 'Lignin biosynthesis' (AraCyc) were the top scoring pathways found in all four data sets in accordance with the actual biochemical function of CYP73A5 [39]. Not only genes of different branches of the downstream phenylpropanoid pathways, but also isoforms for all upstream steps in the shikimate pathway [30] leading to phenylalanine biosynthesis are co-expressed, thus reconstituting the full pathway (Additional File 4).

**Figure 5:**
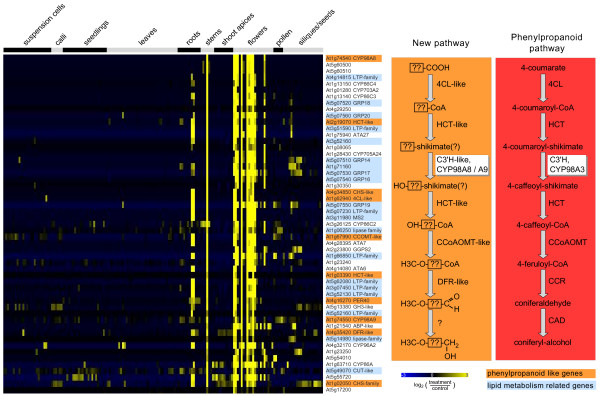
**Expression analysis using CYP98A8 as bait**. Data from published Affymetrix microarrays representing 167 organ and tissue samples were retrieved from the Genevestigator database [10]. Background correction and ratio log_2_-ratio generation was performed as describe in Methods. The expression vector of *CYP98A8 *was compared to those of 4,119 genes annotated in diverse databases to be involved in any metabolic pathway using the 'ExpresionAngler' algorithm [9]. Expression profiles of co-expressed genes with a correlation coefficient of more than 0.6 are shown as a heatmap. Groups of samples are indicated on top of the heatmap. Mean-centered signal intensity ratios are color coded as indicated on the bottom of the heatmap. Genes with similarity to enzymes of the phenylpropanoid pathway are highlighted in orange. Genes related to lipid metabolism are highlighted in blue. Detailed information on the co-expressed genes and samples can be found in Additional File 5. To the right a section of the phenylpropanoid pathway is outlined in red and the putative duplicated pathway as hypothesized based on the co-expression analysis of CYP98A8 is outlined in orange.

It is important to note that a significant proportion of P450s might act in biochemical pathways not yet elucidated and may produce natural compounds which were never described. Obviously, genes in such unknown pathways have not been annotated, and it is therefore impossible to predict these pathways using the co-expression approach. However, even in such cases valuable information can be obtained by careful inspection of co-expressed genes. This may be exemplified using the CYP98 family. *CYP98A3 *encodes *p*-coumaroyl shikimate/quinate 3'-hydroxylase (C3'H) and is involved in the biosynthesis of monolignols [40,41]. This gene is tightly co-expressed with *C4H *and most other characterized genes involved in the general phenylpropanoid pathway (Additional File 4). Two other genes of the same family (*CYP98A8 *and *CYP98A9*) share extensive sequence similarity with *CYP98A3*, but were shown not to encode C3'H [41]. Both *CYP98A8 *and *CYP98A9 *share an overlapping expression pattern that is very distinct from *C3'H*, with expression predominantly in floral tissues (Figure 5 & Additional File 5). In the organ data set, the top scoring co-expressed pathway for both genes appears as 'miscellaneous acyl lipid metabolism' (AcylLipid) due to a large number of putative and known genes related to fatty acid metabolism, which are likely involved in pollen coat/wall development. However, several genes related to the phenylpropanoid pathway are also co-expressed with *CYP98A8 and CYP98A9 *(highlighted in orange in Figure 5). Altogether, they encode 'phenylpropanoid-like' enzymes with unknown functions sharing sequence similarities with characterized phenylpropanoid enzymes [30,32,35]. This co-expression group thus appears to result from the duplication of at least a portion of the phenylpropanoid pathway and its subsequent recruitment for a novel flower specific pathway (Figure 5). Identification of the substrate(s) of any of these enzymes should lead to the elucidation of this 'phenylpropanoid-like pathway'.

In summary, these examples show that co-expression analysis combined with pathway mapping of co-expressed genes is a powerful tool to identify genes encoding enzymes acting in the same biochemical pathway. As a proof of concept, the majority of known P450s were placed in the expected pathway. But the approach also provides leads to novel pathways for a large set of orphan P450s.

### P450s related to plastidial activity (chlorophyll/carotenoid pathways)

One of the most striking features revealed by the co-expression analysis is an unexpectedly large subset of P450 genes being mapped to pathways identified as 'plastidial isoprenoids' (BioPath), 'photosystems' (BioPath), 'photosynthesis' (KEGG or FunCat), and 'biogenesis of the chloroplast' (FunCat). At the 'CYPedia' homepage follow the link 'browse pathways' and 'CYP => pathway' to the corresponding database for detailed information. Their pathway predictions scores, frequently far above 500, are the highest of the whole analysis. Those include *CYP97A3 *and *CYP97C1 *that were recently shown to be involved in the hydroxylation of the β- and ε-rings of carotenoids [42,43], but also as many as 79 other still orphan P450 genes.

**Figure 6:**
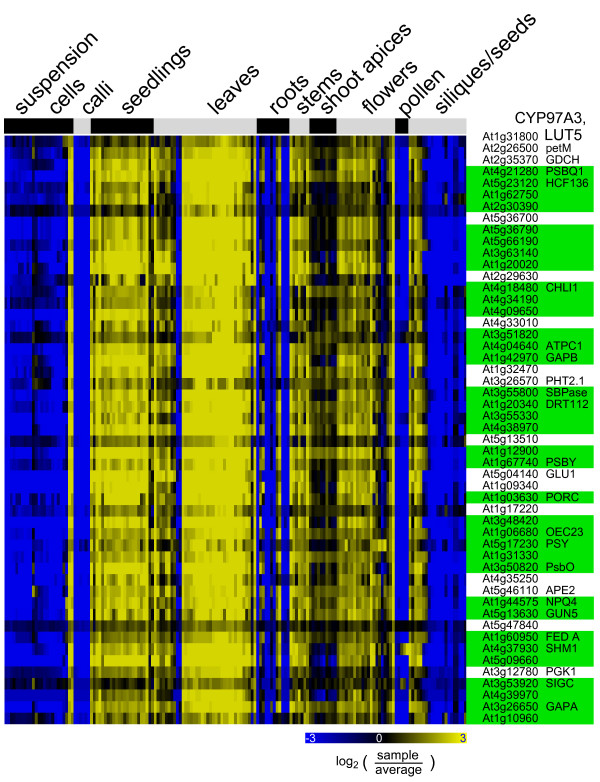
**Co-expression analysis of a P450 associated with plastidial activity: CYP97A3**. Microarray expression data were retrieved from the 'Genevestigator' database and processed as described in Methods. The organ expression vector of *CYP97A3 *was used as bait for co-expression analysis as described in Figure 4. The expression vector of the bait *CYP97A3 *(first row) is shown across 167 organ and tissue samples. 50 co-expressed genes having a correlation coefficient of r > 0.84 are shown in subsequent rows. The resulting heatmap is color coded as indicated. Highlighted in green are genes from the categories 'plastidial isoprenoids' (BioPath), 'photosystems' (BioPath), 'photosynthesis' (KEGG or FunCat), and 'biogenesis of the chloroplast' (FunCat). Detailed information on the co-expressed genes and samples can be found in Additional File 6. Up to 80 additional P450s in the CYPedia analysis share a similar expression profile and pathway prediction.

All these genes show very similar expression patterns, as exemplified in Figure 6 (see also Additional File 6) for *CYP97A3*, with very high expression in all green tissues. They also frequently show down-regulation upon pathogen attack in leaf tissues (not shown). Eleven of them are predicted to have a plastidial localization based on a ChloroP prediction. Based on manual assessment, Schuler and co-workers identified eleven P450s to be likely localized to the plastids [2]; seven of these are among the group with predicted plastidial activity. This may suggest that the role of P450 oxygenases in the metabolism of plastidial (di)terpenoid derivatives, such as carotenoids, chlorophyll prosthetic group, tocopherols, phyllo- and plastoquinones, was so far overlooked. It may also indicate that a number of plant P450 enzymes have functions related to primary photosynthetic metabolism for the synthesis of antioxidants, plastidial structural components, signaling molecules related to energetic metabolism or light perception. The latter case is illustrated by *CYP90A1 *that shows the typical expression pattern depicted in Figure 6. CYP90A1 catalyzes the 23-hydroxylation step in the biosynthesis of brassinosteroids [44] and was recently reported to be under diurnal light-dependent control [45]. On the other hand, some P450 in this group may have house-keeping function or be involved in the biosynthesis of constitutive natural products, which are spatially and temporally coupled to energy production and active plant growth. *CYP86A2*, which was recently described as involved in the biosynthesis of cuticular lipids [21], may be representative of this latter category.

### Candidate P450s acting on triterpenoid compounds

Terpenoids are C5 isoprene-derived compounds which form the largest and most diverse class of natural products. In plants, they play important roles in development and adaptation via hormones and antioxidants, but most of them are mediators of antagonistic or beneficial interactions with other organisms, such as defense against pathogens or attraction of pollinating insects [46]. Among these, triterpenes are produced from 2,3-oxidosqalene by triterpene synthases (TTPS) encoded by 13 genes (including the sterol cyclases CAS and LAS) in *A. thaliana *[47]. Each TTPS produces a unique set of terpenoids, which may then be further modulated, e.g. hydroxylated, by P450s to generate the plethora of decorated triterpenoid compounds. While many *TTPS *genes have been characterized, only one P450 involved in triterpenoid modification has been identified [48]. Our pathway mapping approach identified 63 P450s as co-expressed with genes placed in the category 'triterpene, sterol, and brassinosteroid metabolism' (LitPath) among them 27 belonging into the category 'triterpene biosynthesis' (from the 'CYPedia' homepage follow the link 'browse pathways' and 'pathway => CYP' to 'LitPath'). In order to further identify individual pairs of *TTPS *and *P450 *genes possibly acting in concert, we calculated, for each expression data set, correlation coefficients comparing expression vectors of each *TTPS *with each P450. For seven of the *TTPS *genes, up to six tightly co-expressed P450s (r > 0.75) were identified (Table 2). A total of 20 P450s (represented by 18 probe sets) are co-expressed with at least one *TTPS *in at least one of the datasets. None of these P450s has been characterized to date. Seven of these belong to the *CYP705 *family, while no other family is represented by more than two co-expressed genes, indicating a particular role for this family in triterpenoid modulation, which may be driven by *CYP705*/*TTPS *co-evolution.

**Table 2:** Pearson correlation coefficients comparing expression vectors of triterpene synthase (TTPS) genes with P450s

			r-values			
			
Name	Description	Co-expressed P450	Organ	Stress	Hormone	Mutant
TTPS1	multiproduct triterpene synthase(At1g78500/263123_at)	CYP81D6 or D7 (At2g23220 or At2g23190, 245072_s_at)	**0.91**	0.07	0.01	**0.95**
		CYP705A23 (At3g20140, 257114_at)	**0.50**	0.01	**0.91**	**0.71**
		CYP72A9 (At3g14630, 258111_at)	**0.87**	0.08	-0.07	0.22
		CYP702A1 (At1g65670, 264634_at)	**0.83**	0.03	-0.13	-0.49
		CYP81D2 (At4g37360, 253091_at)	**0.82**	-0.15	-0.03	0.32
		CYP709B1 (At2g46960, 266736_at)	**0.75**	0.03	0.04	**0.54**
TTPS2	arabidiol synthase	CYP705A25 (At1g50560, 261878_at)	**0.80**	0.28	**0.65**	-0.08
	(At4g15340/245258_at)	CYP705A27 (At1g50520, 261879_at)	**0.77**	0.38	0.25	0.20
TTPS3	2,3-oxidosqualene cyclase-like	CYP702A2 (At4g15300, 245547_at)	**0.88**	**0.75**	0.09	0.07
	(At4g15370/245553_at)	CYP705A2 (At4g15350, 245551_at)	**0.82**	**0.61**	0.26	-0.16
MRN1,	marneral synthase	CYP705A12 (At5g42580, 249202_at)	**0.82**	0.36	**0.67**	**0.78**
TTPS5	(At5g42600/249205_at)	CYP71A16 (At5g42590, 249203_at)	**0.80**	0.42	**0.73**	**0.74**
TTPS6	thalianol synthase	CYP705A5 (At5g47990, 248727_at)	**0.92**	**0.55**	**0.89**	**0.84**
	(At5g48010/248729_at)	CYP708A2 (At5g48000, 248728_at)	**0.92**	**0.77**	**0.90**	**0.87**
		CYP71A16 (At5g42590, 249203_at)	**0.83**	0.44	**0.60**	**0.81**
		CYP705A12 (At5g42580, 249202_at)	**0.77**	0.31	**0.55**	**0.68**
LS1	multiproduct triterpene synthase (At1g66960/255912_at)	CYP89A7/A4 (At1g64930/At2g12190, 262865_at)	-0.02	NA	**0.92**	0.01
TTPS4	2,3-oxidosqualene cyclase-like	CYP716A2 (At5g36140, 249686_at)	0.49	0.30	**0.79**	0.29
	(At5g36150/249687_at)	CYP716A1 (At5g36110, 249684_s_at)	0.26	0.21	**0.78**	0.12
		CYP722A1 (At1g19630, 261134_at)	-0.10	0.08	**0.76**	0.07

For each TTPS, all P450s are shown that have an r-value of more than 0.75 in at least one data set with the given TTPS.

**Figure 7:**
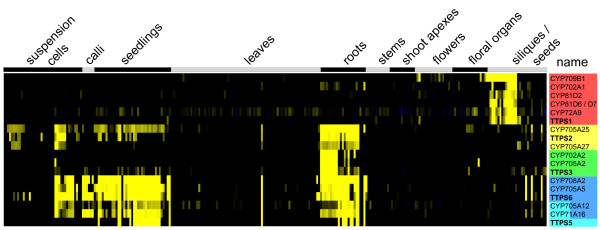
**Organ expression of co-expressed triterpene synthases (TTPS) and P450s**. Microarray expression data were retrieved from the 'Genevestigator' database and processed as described in Methods. Expression vectors from the organ and tissue data sets of five *TTPS *genes (from *A. thalaina *was used as a bait for co-expression analysis comparing it's expression with that of all P450 genes. We retained five TTPS genes, which were co-expressed (r > 0.75) with at least one P450 in the organ and tissue expression data set, and the corresponding P450s (Table 2). This set of genes was used visualize expression. *TTPS *(in bold) and correlated *P450 *genes with high correlation coefficients are color coded.

The strongest correlations were found for *TTPS6 *and *TTPS5 *(*MRN1*). TTPS6 (thalianol synthase) catalyzes the cyclization of 2,3-epoxysqualene to form the tricyclic triterpene thalianol [49], while MRN1 catalyzes an atypical epoxysqualene cyclization into a monocyclic iridal triterpene named marneral [50]. Neither product nor further metabolites have yet been identified *in planta*. Related iridal triterpenoids were however described in *Iridaceae*. *MRN1 *and *TTPS6 *share an overlapping expression pattern with the same set of four P450s in all data sets, though most pronounced in the organ data (Figure 7, Additional File 7). They are highly expressed in roots, seedlings (potentially the root part thereof), and some cell cultures. Within the cluster, *CYP705A5 *and *CYP708A2 *are expressed more similar to *TTPS6*, while *CYP705A12 *and *CYP71A16 *share a more similar organ pattern with *MRN1*, being expressed mainly in more mature root samples (Figure 7). Likewise, this gene set forms a separate cluster in the hormone data set, with induced expression upon cytokinin (zeatin) and MeJ treatments, with again the same sub-clustering (Additional File 7). *MRN1 *is not stress responsive (and therefore having no co-expressed P450s in the stress data set), but *TTPS6*, *CYP705A5 *and *CYP708A2 *form a clear cluster characterized by induced expression in roots upon wounding, drought, and some other stressors, although r-values are comparatively low (Table 2, Additional File 7). The whole group forms again a strong cluster in the mutant data set with a typical expression pattern. It displays repressed expression in *det2 *and *ga1 *mutants (Additional File 7), which are blocked in the biosynthesis of brassinosteroids and gibberellic acid, respectively [51,52]. It appears thus that blockage of hormone pathways branching upstream of TTPS action results in down-regulation of these pathways as well.

In summary, two subgroups of strongly co-regulated genes were identified. Among them, CYP705A5 and CYP708A2 are good candidates for catalyzing further modifications, possibly sequential hydroxylations, of thalianol to form a stress responsive, root specific triterpenoid. While this manuscript was under evaluation, this prediction was fully confirmed by the functional characterization of the thalianol pathway by Fields and Osbourn [53]. Their characterization of this pathway was guided by an operon-like physical clustering of the co-expressed genes. CYP705A12 and CYP71A1, on the other hand, are more likely involved in modifications of marneral to form a triterpene iridoid derivative, similar to multi-hydroxylated iridoids so far considered as characteristic of *Iridaceae *[54]. Equally consistent leads were obtained from the clustering analysis of other P450s related to triterpenoid pathways initiated by TTPS1, TTPS2, and TTPS3.

### P450s related to plant hormone biosynthesis

Cytochrome P450s play central roles in the metabolism of all classes of plant hormones [4]. Our co-expression approach was in particular successful in the case of the octadecanoid pathway leading to the biosynthesis of jasmonate and other oxylipins. Jasmonate is a well characterized stress response signal that also fulfills hormonal actions in stamen and pollen development [55]. Both characterized P450s acting in this pathways, allene oxide synthase (*AOS*, *CYP74A*) and hydroperoxide lyase (*HPL1*, *CYP74B2*) [19], were correctly placed in the pathways 'jasmonic acid biosynthesis' (TAIR-GO) and 'lipoxygenase pathway' (AraCyc), respectively. However, additional P450s might be involved in the metabolism of jasmonate (e.g. catalyzing hydroxylations of jasmonate) and other oxylipins. In addition, a subset of genes involved in defense or plant development is expected to be selectively activated by the jasmonate cascade. Indeed, as many as ten additional P450s are co-expressed with genes related to jasmonate signaling (i.e. being placed into the categories 'jasmonic acid biosynthesis' [AraCyc], 'jasmonic acid biosynthesis' [TAIR-GO], or 'response to jasmonic acid stimulus' [TAIR-GO]). Table 3 lists correlation coefficients with jasmonate related genes for P450s, which have co-expressed gene in at least two data sets, and which have more than five co-expressed genes in at least one data set. Four so far uncharacterized P450s share a common hormone-response profile with many jasmonate related genes (top of Table 3), due to a strong and specific induction upon methyljasmonate treatment. These genes also share a common profile with jasmonate related genes in other datasets (Table 3). Phylogeny and *in vitro *functional analysis predicts most of them (CYP94s, CYP96A4) to be involved in the metabolism of oxylipins [56]. For a second group of genes, correlated expression with the jasmonate pathway is especially striking in the organ data set (bottom of Table 3). Those are known or predicted to participate in the light perception/plastidial activity (CYP97B3, CYP90A1[44], CYP72A11), or the biosythesis of glucosinolates (CYP83B1 [57], CYP71B7). It is interesting to note that in the case of 12-oxophytodienoate reductases, *OPR3 *is co-expresssed with most of P450s (including *AOS *and *HPL1*) in the hormone and stress data sets, while *OPR2 *shares a similar expression with P450s exclusively in the organ data set.

**Table 3:** Pearson correlation coefficients comparing expression vectors of jasmonate related genes

P450 name	Data set	Correlation coefficient (r-value) of P450 with jasmonate related gene													
		
		LOX2	LOX3	LOXL1	LOXL2	AOS	HPL1	AOC1	AOC2	AOC4	OPR2	OPR3	OPRL1/2	TAT3	JR2
CYP74A	organs	0.64	-	-	-	1.00	-	0.88	-	-	0.56	0.69	-	0.60	0.70
AOS	stress	0.67	0.58	-	-	1.00	0.60	0.81	-	-	-	0.71	-	-	-
	hormones	0.57	0.81	-	0.79	1.00	0.59	0.80	0.80	-	-	0.86	-	0.71	0.55
	mutants	-	0.61	0.50	-	1.00	-	0.62	-	-	-	-	-	-	0.51
CYP81D1	organs	-	-	-	-	-	-	-	-	-	-	-	0.55	0.66	-
	stress	0.52	-	-	-	0.57	0.51	0.50	-	-	-	0.60	-	0.61	0.53
	hormones	-	0.51	-	-	0.52	-	0.63	-	-	-	0.61	-	0.54	-
CYP94C1	stress	-	0.58	-	-	-	-	-	0.67	-	-	0.61	-	-	-
	hormones	-	0.79	-	0.79	0.69	-	0.58	0.63	-	-	0.79	-	0.55	-
	mutants	-	0.69	-	0.71	-	-	-	0.62	-	-	0.61	-	-	-
CYP94B1	hormones	-	0.64	-	0.61	0.61	0.53	0.51	-	-	-	0.64	-	-	-
	mutants	-	0.62	-	0.51	0.63	-	0.51	-	-	-	0.60	-	-	-
CYP96A4	organs	0.60	0.56	-	0.50	0.64	0.52	0.61	-	-	-	0.61	-	0.65	0.57
	hormones	-	0.64	-	0.60	0.74	0.71	0.60	-	-	-	0.72	-	-	-
CYP74B2	organs	0.73	-	-	-	-	1.00	-	-	0.52	-	-	-	-	0.62
HPL1	stress	0.67	-	-	-	0.60	1.00	0.53	-	-	-	0.57	-	-	0.54
CYP97B3	organs	0.71	-	-	-	0.58	0.59	0.63	-	-	-	-	0.66	-	0.65
CYP90A1	organs	0.63	-	-	-	0.56	-	0.57	-	0.66	-	-	0.51	-	0.56
CYP72A11	organs	0.74	-	-	-	0.69	-	0.78	-	0.53	-	0.56	0.65	0.52	0.67
CYP83B1	organs	-	-	-	-	0.76	-	0.69	-	-	0.57	0.63	-	0.58	0.55
CYP71B7	organs	-	-	-	-	0.80	-	0.82	-	-	0.61	0.52	-	0.52	0.56
CYP72A8	organs	0.59	-	-	-	0.56	-	0.62	-	-	0.60	-	0.68	0.59	-

Shown are genes in the pathways, which have at least one co-expressed P450; only P450s are shown, which have at least six co-expressed jasmonate genes (r > 0.5) in at least one data set. Abbreviations of genes (AGI locus and Affymetrix probe set are given in brackets): LOX2: lipoxygenase 2 (At3g45140, 252618_at); LOX3: lipoxygenase 3 (At1g17420; 261037_at); LOXL1: lipoxygenase like 1 (At1g67560; 260190_at); LOXL2: (At1g72520; 260399_at); AOS, CYP74A: allene oxide synthase (At5g42650; 249208_at); HPL1, CYP74B2: hydroperoxide lyase 1 (At4g15440; 245253_at); AOC1: allene oxide cyclase 1 (At3g25760; 257641_s_at); AOC2: allene oxide cyclase 2 (At3g25780; 257644_at); AOC4: allene oxide cyclase 4 (At1g13280; 259366_at); OPR2: 12-oxophytodienoic acid reductase 2 (At1g76690; 259875_s_at); OPR3: 2-oxophytodienoate reductase 3 (At2g06050; 265530_at); OPRL1/2: 12-oxophytodienoate reductase like 1 and 2 (At1g17990/At1g18020; 255895_at); TAT3: tyrosine aminotransferase 3 (At2g24850; 263539_at); JR2: cystine lyase, jasmonic acid response 2.

Distinct P450 subsets were associated with various other hormone pathways (not shown). However, r-values are generally lower compared to the jasmonate related genes and co-expression is limited to fewer genes in the respective pathways. This may be due to the fact that metabolism of these hormones is less characterized, or, more likely, due to the relatively low and cell/tissue specific expression of most of the genes involved in these hormonal pathways.

In summary, the co-expression approach associates groups of P450s with specific hormonal pathways. The analysis is however more informative in the case of stress signaling which involves strong responses than in the case of low concentration hormones controlling plant development. It is thus expected to mainly support characterization of new stress signaling pathways.

## Conclusion

The abundance of publicly available microarray expression data provides a stunning amount of information that has been exploited only sparsely to date. A correlation between gene expression and their biological/biochemical roles is necessary, and when genes encode metabolic enzymes acting in the same pathway, they are expected to be co-regulated. The data presented here covering known pathways largely confirm these assumptions. Based on co-expression analysis of the complete P450 superfamily in *A. thaliana *we have generated novel hypotheses regarding biochemical and biological functions for a large number of individual genes or gene groups involved in common pathways. Strikingly, the first validation of a new pathway predicted from our data was published independently during evaluation of this manuscript [53], thus further confirming the potential of this approach. More leads will emerge from this analysis in the next years, supported by an increasing number of characterized genes functions. New hypotheses can now be addressed experimentally by exploiting the expanding toolbox of reverse genetics, such as insertion mutants combined with targeted metabolic profiling, and by reverse biochemistry using collections of recombinant proteins and medium throughput screening of substrate collections [58]. The same approach can also be extended to other gene families, including transcription factors, and thus has the potential to considerably accelerate the molecular understanding of plant natural product metabolic networks and regulation.

## Materials and Methods

### Probe set selection and expression data of P450 genes

A collection of all cytochromes P450 from *A. thaliana *(271 genes as of April 2005) and the corresponding AGI (Arabidopsis Genome Initiative) locus identifiers (Atxgxxxxx) were retrieved from the' PlaCe Arabidopsis P450 database' (Table 2). For 21 P450 genes annotated at PlaCe, no AGI locus was associated. Those included 18 annotated pseudogenes. Two pairs of P450 genes were associated with the same AGI-locus (*CYP71A27P *and *CYP71A28*: At4g20240; *CYP71A23 *and *CYP71A24*: At3g48290), leaving a total of 248 AGI loci. These were used to identify corresponding probe sets on the Affymetrix ATH1 microarray using the 'Genevestigator' probe selection tool [10]. 21 genes were not represented on the array. The remaining 227 genes were represented by a total of 229 probe sets, with 26 genes being represented by more than one probe set, and 32 probe sets representing more than one gene. Using the 'Genevestigator' probe selection tool we identified all genes recognized by these probe sets, and if more than one probe set was present for a given gene, we selected a single, specific (if available) probe set for that gene. This resulted in 216 selected probe sets; of these 191 recognize a single P450 gene, 21 recognize two genes, 3 probe sets may hybridize with three genes and one recognizes four genes for a total of the 227 represented P450s, and three non-P450 genes (flanking genes that are also recognized by the probe set). The probe sets used and the genes recognized by these probe sets can be found at the 'CYPedia' home page.

We then retrieved normalized expression data for these probe sets from the 'Genevestigator Digital Northern' tool [10]. Data were downloaded in May 2005 (dataset 1), covering 1,823 microarray experiments, and in April 2006 (dataset 2, an update including dataset 1) covering 2,202 microarrays. For each probe set, background was defined as the average signal intensity of all probes called 'absent' by the Affymetrix software, and all absent probes were set to this background value. If replicate arrays were available, the mean intensity of all replicates was determined. Each experiment was placed in one of the following four categories: i) organ and tissue samples from wild type plants, ii) stress treatment of wild type plants, iii) hormone, nutrient (deprivation), and other treatments of wild type plants, and iv) mutant plants compared to wild type samples treated equally (if applicable). Signal intensities from organ and tissue samples were then compared to the background intensities, thus generating log_2_-ratios over background. Intensities from both treatment groups were compared to signal intensities from the corresponding control samples generating log_2_-ratios comparing treatment with control, and intensities from mutant samples were compared with intensities from equally treated wild type samples thereby generating log_2_-ratios for mutants compared to wild-type. Each dataset was divided into 30 expression groups using K-means clustering and the combined heatmaps from all clusters can be found at the 'CYPedia' home page following the link 'view matrices'. For visualization of the expression matrices the 'HeatMapper' tool at the 'Bio-Array Resource (BAR)' [9] was used and the resulting heatmaps were incorporated into commonly used spreadsheet formats (Adobe PDF, Microsoft Excel and OpenOffice Calc).

### Selection of metabolic genes

A list of genes related to any aspect of plant metabolism (pathway database) was generated by retrieving all *A. thaliana *genes, which were annotated in the following databases: i) 'KEGG Orthology (KO) – Arabidopsis thaliana' (KEGG) [59], ii) the 'Metabolic Pathways' at 'The Arabidopsis Information Resource' (AraCyc) [60]; iii) the 'Arabidopsis Lipid Gene Database' (AcylLipid) [61], iv) the 'Biochemical Pathway Knowledge Database' (BioPathAt) [34], v) a selection of publications devoted to the annotation of secondary metabolic pathways (Litpath) [30-33,35,62]. Information from all databases were combined in one data matrix and Affymetrix probe sets were selected for the set of unique genes as described above resulting in 4,129 unique probe sets. For this set of genes, annotations were added that were derived from the 'Functional Catalogue' at the 'Munich Information Center for Protein Sequences (MIPS-FunCat) [36] and manually curated 'GeneOntology' terms from TAIR [63] (i.e. having the evidence codes IDA [inferred from direct assay], IMP [inferred from mutant phenotype] and/or TAS [traceable author statement].

Each gene was given a pathway annotation score with: ten points for biochemically characterized genes (i.e. annotation as 'functional' in 'AcylLipid' or 'BioPath', or identified in literature reviews); nine points for genes with immediate biochemical function described as IDA in TAIR-GO, eight point for genes annotated as 'functional(?)' or 'inferred from mutant phenotype' in 'AcylLipid', 'BioPath', or literature; seven points for genes with evidence code IMP at TAIR-GO; six points for genes with a described mutant phenotype, but with unclear molecular function; five points for genes with high similarity (WU-BLAST e < 10^-50^) to a characterized plant gene; four point for genes with high similarities to another plant gene, but function of that gene not validated; three points for genes with similarity (WU-BLAST 10^-10^e < 10^-50^) to a characterized plant gene; two points for genes with low similarities (WU-BLAST e > 10^-10^) to a characterized plant gene; one point for members of large gene families with low similarities (WU-BLAST e > 10^-10^) to a characterized plant gene.

### Co-expression analysis and pathway mapping

Affymetrix expression data for the selected 4,129 probe sets were retrieved and processed as described above for the P450s and the expression matrices were merged. Co-expression analysis was performed as described earlier [9]. In brief, expression vectors were mean-centered and Pearson correlation coefficients (r-values) were calculated between the expression vector of each P450 and those of the 4,129 genes in the "pond" for each data set. Subsequent manipulations were performed using the R environment [64]. For each P450 and data set co-expressed genes with r > 0.5 were retrieved and the corresponding biochemical pathways were extracted from the pathway database (see above). For each pathway, the number of co-expressed genes was counted and the sum of annotation scores (see above) was calculated. The pathway was retained only when at least one gene in the list had more than six annotation points. The number and the score of co-expressed genes in a given pathway was compared to the total number and score of all genes in that pathway. Based on a tailed hypergeometric distribution analysis only pathways over-represented in the group of co-expressed genes (p [hyper] < 0.005) were retained. Subsequently, pathways identified in all four datasets were identified and the number and scores of genes found in each dataset were summed. The resulting tables were sorted according to scores and imported into an OpenOffice Calc (OpenOffice.org) template and thumbnails of the actual expression heatmaps, generated using the 'Heatmapper plus' tool at the 'BAR' [9], were added and saved in html format. Results for each P450 can be found at the 'Pathway Map' webpage for each P450. Expression data and pathway information data for co-expressed genes (r > 0.5 for a maximum of 50 genes) were merged and sorted according to r-value. Expression tables were color coded using the 'Heatmapper plus' tool at the 'BAR' and saved as static web pages linked to the corresponding pathway maps.

### Array platform comparison

P450 expression data generated using a spotted microarray covering gene specific PCR products were retrieved from the 'Functional Genomics of Arabidopsis P450s' web page (Table 1). Using this dual channel platform (CYP-array), signal intensities in roots from 1 week old seedlings (and four other organs) were generated by comparison to a 'universal RNA' sample. This 'universal RNA' consists of a mixture of RNAs derived from roots and shoots from seedlings and leaves, stems and flowers from mature plants [2]. In order to generate a similar 'universal control' from public ATH1 microarrays, we selected 14 shoot samples from seedlings, 9 leaf samples from mature plants, 17 root samples from seedlings, 19 whole flower samples, and 10 stem samples from the processed organ data set (see above). We then calculated the mean log_2 _intensities over background form all samples and compared it to the mean intensity of the root samples and thereby created root/'universal control' ratios similar to those from the CYP-array. For the latter, not detectable intensities were artificially set to a ratio of 0.05 compared to the universal control and ratios were log_2_-transformed. Expression data for genes represented on both platforms were mean centered across the experiments. Based on a linear regression model comparing the two data sets an R^2 ^value was calculated.

## Availability and requirements

CYPedia: 

## Authors' contributions

JE analyzed the microarray data, and designed and built the 'CYPedia' database. VS and AO helped building the web interface. JFG was/is involved in updating the database. NJP performed the co-expression analysis. DWR and JE conceived of the project. DWR directed the study and helped with interpretation of data. JE and DWR wrote the manuscript. All authors read and approved the final manuscript.

## Supplementary Material

Additional File 1 **Locus and probe set information for P450s**. Given are the Affymetrix AtH1 microarray probe sets used for cytochromes P450 and the name and AGI loci recognized by these probe sets. In addition, the number of experiments in the respective data sets with detectable expression (more than twofold difference from the control) is given, as well as the fraction of samples with detectable expression. In the organ data sets control is defined for each probe set as the average signal intensity on arrays were this probe set was called 'absent' by the Affymetrix software. In the stress and hormone data sets control is defined as the signal intensities of untreated control samples. In the mutant data set control is defined as the signal intensities in the corresponding wild type samples. Click here for file

Additional File 2 **Stress responsive expression of P450s**. Microarray expression data were retrieved from the 'Genevestigator' database and processed as described in Methods. Only genes that are up-regulated (>twofold) in more than 30% of at least one treatment group as indicated on top were selected. Background corrected expression intensities were compared to untreated control experiments and log_2_-ratios were used for hierarchical cluster analysis with complete linkage. The resulting heatmap is color coded as indicated in the overview image in Sheet 1 (overview). Details on the individual samples can be found in Sheet2 (details) of this spreadsheet. Click here for file

Additional File 3 **Pathway predictions based on co-expression analysis of P450s with known functions**. Top scoring co-expressed pathways for P450s with characterized biochemical functions. Click here for file

Additional File 4 **Co-expression analysis using CYP73A5 encoding cinnamate 4-hydroxylase as bait**. Data from published Affymetrix microarrays (representing a) 167 organ and tissue samples and b) 243 stress related treatments) were retrieved from the Genevestigator database [10]. Background correction and ratio log2-ratio generation was performed as describe in Methods. The expression vectors of CYP73A5 were compared to those of 4,119 genes annotated in diverse databases to be involved in any metabolic pathway using the 'ExpresionAngler' algorith [9]. Expression profiles of co-expressed genes with a correlation coefficient of more than 0.5 are shown as a heatmap. Groups of samples are indicated on top of the heatmap. Mean centred signal intensity ratios are colour coded as indicated on the bottom of each heatmap. Genes encoding enzymes of the phenylpropanoid and shikimate pathways are highlighted in red and green, respectively. Sheet 1 shows overview image, detailed information on the co-expressed genes and samples can be found in sheets 2 (organs) and 3 (stress) of this file. Click here for file

Additional File 5 **Co-expression analysis using CYP98A8 as bait**. Data from published Affymetrix microarrays representing 167 organ and tissue samples were retrieved from the 'Genevestigator' database [10]. Background correction and ratio log_2_-ratio generation was performed as describe in Methods. The expression vector of CYP98A8 was compared to those of 4,119 genes annotated in diverse databases to be involved in any metabolic pathway using the 'ExpresionAngler' algorithm [9]. Expression profiles of co-expressed genes with a correlation coefficient of more than 0.6 are shown as a heatmap table. Brief descriptions of the experiments and the experiment identifier from the 'Genevestigator' database are indicated on top of the heatmap. Mean-centred signal intensity ratios are colour coded as indicated to the right. Click here for file

Additional File 6 **Co-expression analysis using CYP98A8 as bait**. Data from published Affymetrix microarrays representing 167 organ and tissue samples were retrieved from the Genevestigator database [10]. Background correction and ratio log2-ratio generation was performed as describe in Methods. The expression vector of CYP97A3 was compared to those of 4,119 genes annotated in diverse databases to be involved in any metabolic pathway using the 'ExpresionAngler' algorithm [9]. Expression profiles of co-expressed genes with a correlation coefficient of more than 0.84 are shown as a heatmap table. Brief descriptions of the experiments and the experiment identifier from the Genevestigator database are indicated on top of the heatmap. Mean-centered signal intensity ratios are color coded as indicated to the right. This table corresponds to Figure 6. Click here for file

Additional File 7 **Cluster analysis of triterpene synthases (TTPS) and P450s**. Microarray expression data were retrieved from the 'Genevestigator' database and processed as described in Methods. Expression vectors from the four data sets of all twelve TTPS genes from *A. thaliana *were used as bait for co-expression analysis comparing its expression with that of all P450 genes. We retained seven TTPS genes, which were co-expressed (r > 0.75) with at least one P450 in at least one of the four expression data sets and the corresponding P450s (Table 2). This set of genes was used for hierarchical clustering with complete linkage in a) the organ expression data set and b) the mutant data set as shown in the overview image in Sheet 1. TTPS and clusters with P450 genes with high correlation coefficients are colour coded. Detailed information on the co-expressed genes and samples can be found in sheets 2 (organs), 3 (stress), 4 (hormones), and 5 (mutants) of this file. The numbers in brackets refer to the experiment ID from the Genevestigator database. Click here for file
