# Supplementary material for: An extensive (co-)expression analysis tool for the cytochrome P450 superfamily in Arabidopsis thaliana
Source: BMC Plant Biol. 2008 Apr 23;8:47. doi: 10.1186/1471-2229-8-47 (PMC2383897; doi:10.1186/1471-2229-8-47)
Supplement: Additional File 3 — Pathway predictions based on co-expression analysis of P450s with known functions. Top scoring co-expressed pathways for P450s with characterized biochemical functions. [file 1471-2229-8-47-S3.pdf]

Additional File 3 Pathway predictions based on co-expression analysis of P450s with known functions

Name	Co-expressed pathway	Source	Known pathway[a]	Description[a]
**P450s with correct prediction:**				
CYP51G1 (CYP51A2)	sterol biosynthesis	BioPath	sterol / steroid metabolism	obtusifoliol 14α-demethylase
CYP701A3, GA3	gibberellin metabolism	LitPath	gibberellin metabolism	multifunctional ent-kaurene oxidase
CYP707A1	abscisic acid biosynthesis	AraCyc	abscisic acid degradation	abscisic acid 8'-hydroxylase
CYP734A1, BAS1	triterpene, sterol, and brassinosteroid metabolism	LitPath	brassinolide degradation	26-hydroxylase for brassinolide and castasterone
CYP73A5, C4H	lignin biosynthesis	AraCyc	phenylpropanoid metabolism, lignin biosynthesis	cinnamic acid 4-hydroxylase
CYP74A, AOS	jasmonic acid biosynthesis	TAIR-GO	oxylipin pathway, jasmonate biosynthesis	allene oxide synthase
CYP74B2, HPL1	lipoxygenase pathway	AraCyc	oxylipin pathway	hydroperoxide lyase
CYP75B1, F3'H	flavonoid biosynthesis	TAIR-GO	flavonoid biosynthesis	flavonoid 3'-hydroxylase
CYP79B3	glucosinolate metabolism	LitPath	indole glucosinolate biosynthesis / camalexin biosynthesis / auxin biosynthesis	conversion of tryptophan to oxime
CYP79F1, SPS, BUS1	glucosinolate metabolism	LitPath	aliphatic glucosinolate biosynthesis	conversion of mono to hexahomomethionine
CYP79F2	glucosinolate metabolism	LitPath	long chain aliphatic glucosinolate biosynthesis	long chain penta and hexahomomethionine in synthesis of long chain aliphatic glucosinolates
CYP83A1, REF2	glucosinolate metabolism	LitPath	aliphatic glucosinolate biosynthesis	oxidation of methionine-derived oximes
CYP83B1, SUR2, RNT1, RED1, ATR4	glucosinolate metabolism	LitPath	indole glucosinolate biosynthesis	oxidation of indole-3-acetyldoxime
CYP84A1, F5H, FAH1	lignin biosynthesis	TAIR-GO	phenylpropanoid metabolism, S-lignin biosynthesis	coniferaldehyde 5-hydroxylase
CYP85A2, BR6OX2	brassinosteroid biosynthesis	TAIR-GO	brassinolide metabolism	brassinosteroid-6-oxidase
CYP86A4	fatty acid elongation and wax and cutin metabolism	AcylLipid	fatty acid metabolism	short-chain fatty acid ω-hydroxylase (lauric acid to ω-hydroxylauric acid)
CYP86A7	fatty acid elongation and wax and cutin metabolism	AcylLipid	fatty acid metabolism	short-chain fatty acid ω-hydroxylase (lauric acid to ω-hydroxylauric acid)
CYP86A8, LCR	fatty acid elongation and wax and cutin metabolism	AcylLipid	fatty acid metabolism	lacerata; ω-hydroxylase for saturated and unsaturated C12 to C18 fatty acids
CYP88A3, KAO1	gibberellin metabolism	LitPath	gibberellin metabolism	multifunctional ent-kaurenoic acid oxidase
CYP90B1, DWF4	triterpene, sterol, and brassinosteroid metabolism	LitPath	brassinosteroid metabolism	steroid C-22 hydroxylase (campestanol to 6-deoxocathasterone)
CYP90C1, ROT3	steroid biosynthesis	TAIR-GO	brassinolide metabolism	steroid C-2α hydroxylase (typhasterol to castasterone)

Additional File 3 Pathway predictions based on co-expression analysis of P450s with known functions

CYP90D1	brassinosteroid biosynthesis	TAIR-GO	brassinolide metabolism	downstream brassinosteroid synthesis, exact substrate not identified
CYP97A3, LUT5	plastidial isoprenoids (chlorophylls, carotenoids, tocopherols, plastoquinone, phylloquinone)	BioPath	carotenoid metabolism	α-carotene β-ring hydroxylase
CYP98A3, C3'H	lignin biosynthesis	AraCyc	phenylpropanoid metabolism, lignin biosynthesis	4-coumaryl shikimic/quinic ester 3'-hydroxylase
**P450s with correct tendency in prediction:**				
CYP79B2	tryptophan biosynthesis	TAIR-GO	indole glucosinolate biosynthesis / auxin biosynthesis / biosynthesis tryptophan derivatives	conversion of tryptophan and tryptophan analogs to oximeesis
CYP97C1, LUT1	chlorophyll biosynthesis	AraCyc	carotenoid metabolism	carotenoid ε-ring hydroxylase
CYP71B15, PAD3	tryptophan biosynthesis	TAIR-GO	camalexin biosynthesis, biosynthesis tryptophan derivatives	conversion of (S)-dihydrocamalexic acid to camalexin
**P450s with no prediction:**				
CYP85A1, BR6OX	no co-expressed pathway	-	brassinolide metabolism	brassinosteroid-6-oxidase
CYP79A2	no co-expressed pathway	-	benzylglucosinolate biosynthesis	conversion of phenylalanine to oxime
CYP72C1, SHK1, SOB7, CHI2	no co-expressed pathway	-	brassinosteroid degradation	controls brassinosteroid (BR) homeostasis by modulating BR concentration
CYP88A4, KAO2	no co-expressed pathway	-	gibberellin metabolism	multifunctional ent-kaurenoic acid oxidase
**P450s with wrong prediction:**				
CYP710A2	γ-hexachlorocyclohexane degradation	KEGG	sterol biosynthesis	Sterol C-22 desaturase (24-epi-campesterol to brassicasterol)
CYP710A1	phenylpropanoid metabolism	BioPath	sterol biosynthesis	Sterol C-22 desaturase (ß-sitosterol to stigmasterol)
CYP711A1, MAX1	core phenylpropanoid metabolism	BioPath	carotenoid metabolism	Biosynthesisi of a likely carotenoid-derived hormone with branch-inhibiting activity
CYP735A1	cell Wall Carbohydrate metabolism	BioPath	cytokinin metabolism	trans-hydroxylase for isopentenyladenine, tri/di/monophosphates
CYP735A2	cell Wall Carbohydrate metabolism	BioPath	cytokinin metabolism	trans-hydroxylase for isopentenyladenine, tri/di/monophosphates
CYP90A1, CPD	chlorophyll biosynthesis	TAIR-GO	brassinolide metabolism	steroid 23α hydroxylase (cathasterone to teasterone)
CYP86A1	stilbene, coumarine and lignin biosynthesis	KEGG	fatty acid metabolism	ω-hydroxylase for saturated and unsaturated C12 to C18 fatty acids
CYP86A2	chlorophyll biosynthesis	TAIR-GO	fatty acid metabolism, cutin biosynthesis	short-chain fatty acid ω-hydroxylase (lauric acid to ω-hydroxylauric acid)
CYP707A4	sterol biosynthesis	BioPath	abscisic acid degradation	abscisic acid 8'-hydroxylase
CYP707A3	secondary metabolism	FunCat	abscisic acid degradation	abscisic acid 8'-hydroxylase
CYP707A2	phenylpropanoid metabolism	BioPath	abscisic acid degradation	abscisic acid 8'-hydroxylase

[a] references can be found at the CYPedia home page (Table 2)
